# Vitreous haemorrhage in massive hemorrhagic polypoidal choroidal vasculopathy: clinical characteristics and surgical outcomes: Vitreous hemorrhage in PCV

**DOI:** 10.1186/s40942-015-0025-4

**Published:** 2015-12-20

**Authors:** Raja Narayanan, Kopal Mithal, Subhadra Jalali, Jay Kumar Chhablani, Annie Mathai, Md Hasnat Ali

**Affiliations:** 1 Srimati Kanuri Santhamma Retina Vitreous Centre, L V Prasad Eye Institute, L V Prasad Marg, Banjara Hills, Hyderabad, Andhra Pradesh 500 034 India; 2 Department of Biostatistics, Kallam Anji Reddy Campus, L V Prasad Eye Institute, Hyderabad, India

**Keywords:** Polypoidal choroidal vasculopathy, Vitrectomy, Vitreous haemorrhage

## Abstract

**Background:**

To report the outcomes of vitreous hemorrhage (VH) associated with hemorrhagic polypoidal choroidal vasculopathy (PCV).

**Methods:**

A retrospective study of 28 eyes of 27 consecutive patients of hemorrhagic PCV with VH, which were managed surgically between January 2003 and December 2011, was performed. All patients underwent pars plana vitrectomy for VH associated with PCV. The main outcome measure was best-corrected visual acuity (BCVA) at baseline, at 1, 3 and 6 months post operatively and at last follow up.

**Results:**

The visual acuity measured on early treatment diabetic retinopathy study (ETDRS) chart improved in 16 eyes (57.1 %) by two or more lines, remained unchanged in nine eyes (32.1 %) and decreased in three (10.7 %) after surgery when compared to baseline VA. The mean baseline VA was 2.69 ± 0.57 logMAR units (<20/2000) which improved to 1.65 ± 0.93 logMAR units (20/800) at 1 month post operative visit and was sustained at 1.72 ± 1.12 (20/800) with an improvement of 0.96 logMAR units (p < 0.001, 95 % CI 0.54–1.37). The average postoperative follow up was for 14.2 months (range 1–84). The complications noted in postoperative follow up were cataract (n = 10), macular scaring (n = 9), organised dehemoglobinised blood (n = 7), retinal tear or detachment (n = 5), recurrent VH (n = 3) and choroidal detachment (n = 1).

**Conclusion:**

Majority of patients with loss of vision due to VH secondary to hemorrhagic PCV have sustained improvement in visual acuity following surgery.

## Background

Polypoidal choroidal vasculopathy (PCV) is a persistent and chronic disease with a variable course [1]. Although the prognosis of PCV is commonly reported to be more favorable and the clinical course more stable as compared with exudative age-related macular degeneration (AMD) [2–4], the natural history of PCV, especially haemorrhagic PCV, is not clearly known and may not be as benign as previously reported [1].

The primary abnormality in PCV involves the choroidal circulation, and the characteristic lesion is an inner choroidal vascular network of vessels ending in an aneurysmal bulge or outward projection seen clinically as reddish orange polyps [5]. Leakage in the vessel wall at dilatations leads to serous pigment epithelial detachments (PED) and lipid deposition in the exudative form of PCV while recurrent serosanguinous detachments of the retinal pigment epithelium and neurosensory retina are the predominant manifestation of hemorrhagic PCV. Massive hemorrhages may develop from rupture of venules and occasionally arteries. Sequelae of this is a sudden onset severe visual loss due to break through vitreous hemorrhage (VH) [1, 6–10].

About half the cases of PCV have a stable course and relatively favorable visual outcome, while the others have persistent leakage, repeated haemorrhages and poor visual outcome [1, 4]. Profound visual loss in hemorrhagic PCV primarily results from recurrent serosanguinous haemorrhagic detachments involving the fovea or recurrent VH.

There is a lack of understanding of hemorrhagic PCV and there are very few reports in the literature on the outcomes of conservative [1, 4], or surgical management of PCV with VH [4, 6]. In the current study we report the incidence, clinical characteristics, management and surgical outcome in haemorrhagic PCV presenting with vitreous haemorrhage.

## Methods

This study was a retrospective, interventional consecutive case series. The study was approved by the local ethics committee of L.V. Prasad Eye Institute (LEC-11-160) and all participants gave consent for using patient data for research purpose. Medical records of all cases having a diagnosis ICD coding of PCV and vitreous haemorrhage who underwent pars plana vitrectomy (PPV) were identified and studied. Twenty-eight eyes of 27 consecutive patients treated surgically for PCV associated with VH were included.

Due to the presence of VH, the media clarity did not allow the visualization of the fundus or angiography in most eyes at presentation. Although the EVEREST trial laid down guidelines for the diagnosis of PCV [11], massive hemorrhagic PCV may not show the same features at presentation in the affected eye. The vitreous haemorrhage was attributed to PCV on the basis of preoperative B-scan features [6], and confirmed with demonstration of polypoidal lesions and/or branching vascular network in the study or fellow eye on indocyanine angiography (ICGA) in the past or post-operatively, as laid down in the EVEREST trial [11]. Patients were excluded if the same or fellow eye had any other vascular or degenerative retinal or macular condition, or any other ocular pathology which could limit their vision.

After a preoperative written informed consent all patients underwent standard 20 or 23 gauge PPV. Removal of subretinal blood was not attempted in this series.

The data pertaining to age, gender, duration of diabetes and hypertension, duration of symptoms, best-corrected visual acuity (BCVA) at presentation, 1, 3, 6 months post-operative and at the last follow up, intraocular pressure (IOP, Goldman applanation tonometry), surgical procedures performed, surgical complications and duration of follow up were collected and are summarized in Table 1.

**Table 1:** Demographic and baseline characteristics

1.	No of eyes	28
2.	Age (mean)	58.89 years (range 36–82)
3.	Gender	22 males, 6 females
4.	Bilateral PCV + VH	4 (14.28 %)
5.	DM/duration	10 patients/13 years (range 3–25 years)
6.	Hypertension/duration	15 patients/10.5 years (range 1–36 years)
7.	Eye	13 OD, 15 OS
8.	Duration of symptoms in months	6.2 (range 1–48 months)
9.	Lens status	11 phakic, 6 pseudophakic, 1 aphakic
10.	Previous treatment	VR surgery (n = 3), IVB (n = 5), PDT (n = 3), Laser (n = 1), IVTA (n = 1), Oral steroids (n = 3)
11.	Fundus exam at presentation	No view (n = 21), hemorrhagic PED (n = 6), subretinal blood (n = 4), pigmentary changes (n = 1)

*PCV* polypoidal choiroidal vasculopathy, *VH* vitreous hemorrhage, *DM* diabetes mellitus, *VR* vitreoretinal, *IVB* intravitreal bevacizumab, *PDT* photodynamic therapy, *IVTA* intravitreal triamcinolone acetonide, *PED* pigment epithelial detachment

The primary outcome measure of this series was the number of patients who improved by two or more lines on early treatment diabetic retinopathy study (ETDRS) chart after surgery. Secondary outcome measures were mean change in logMAR visual acuity, and complications of surgery.

The visual improvement post operatively and the differences between BCVA at 1 month post operative visit and final follow up from the baseline pre operative visual acuity were compared using paired t test. Statistical analysis was done using Statistical software R, version 2.14.1.

## Results

Twenty-eight eyes of 27 patients (21 men, 6 women) with a mean age of 58.89 ± 13.13 years (range 36–82) underwent PPV for vitreous haemorrhage associated with PCV between January 2003 and December 2011. Their baseline demographic characteristics are shown in Table 1. Ten (37 %) patients had a history of diabetes mellitus for an average of 13 years (range 3–25 years) and 16 (59 %) patients had a history of hypertension for an average 10.5 years (range 1–36).

**Fig. 1:**
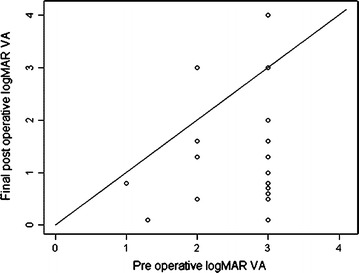
Scatter plot comparing pre-operative and post-operative best-corrected visual acuity

The mean duration of symptoms at presentation was 6.2 ± 3.3 months with sudden onset profound painless diminution of vision being the most common presenting complaint. The mean preoperative baseline logMAR visual acuity was 2.69 ± 0.58 (<20/2000, range 20/400 to perception of light, PL). At 1 month postoperative follow up, the mean BCVA was 1.69 ± 0.93 (20/979, range 20/20 to PL) with a mean improvement of 1.00 logMAR units from the baseline (p < 0.001, 95 % CI 0.64–1.34). At 3 and 6 months follow up the BCVA was 1.46 ± 0.93 (20/576, range 20/20 to PL) and 1.57 ± 1.26 (20/743, range 20/20 to no PL) respectively. The mean BCVA at the last post operative follow up visit was 1.73 ± 1.14 (20/1074, range 20/20 to no PL) with an improvement of 0.96 logMAR units from the baseline (p < 0.001, 95 % CI 0.54–1.37). The BCVA improved by two or more lines on ETDRS chart in 16 of 28 eyes (57.1 %), stabilized or improved by less than two lines in nine (32.1 %) and worsened in three (10.7 %) at a mean follow up of 14.28 months. The three eyes which worsened post-operatively had a preoperative vision of hand movement which decreased to PL in one and loss of PL in the other two eyes. The results of visual outcome have been summarized in Table 2. The trend of BCVA change from baseline preoperative to post operative last visit is shown in Fig. 1.

**Table 2:** Surgical management and postoperative outcome

1.	Baseline BCVA at presentation	2.69 ± 0.57 (<20/2000)
2.	BCVA at 1 months postoperative visit improvement from baseline	1.65 (SD 0.93, 20/800, p = 0.001), range 20/20 to no PL, 0.99 logMAR units, (p = < 0.001, 95 % CI 0.64–1.34)
3.	BCVA at 6 months postoperative visit	1.53 (SD 1.2, 20/600, p = 0.0001), range 20/20 to no PL
4.	Improvement from baseline to last visit	0.96 logMAR units, (p < 0.0001, 95 % CI 0.54–1.37)
5.	Mean follow up	14.8 months (range 1–84 months)
6.	BCVA improved by two lines or more than presenting VA at last follow up	16 of 28 eyes (57.1 %)
7.	BCVA worsened at last follow up than baseline BCVA	3 of 28 eyes (10.7 %)
8.	Complications	Cataract (n = 10), macular scar (n = 4), organised dehemoglobinised blood/exudates (n = 7), retinal detachment (n = 5), iatrogenic retinal breaks (n = 5), recurrent VH (n = 3), choroidal detachment (n = 1)

*BCVA* best-corrected visual acuity, *PL* perception of light, *VH* vitreous hemorrhage

Prior to surgery, five study eyes had been treated with intravitreal bevacizumab, one received intravitreal triamcinolone, three had undergone focal laser treatment, two eyes had undergone sclera buckling in the same eye in the past and three eyes had a history of photo dynamic therapy (PDT). Three patients in whom B-scan showed exudative retinal detachment were treated with oral steroids preoperatively. Nine (33.3 %) patients had bilateral PCV of which four (14.8 %) also had associated VH with PCV in the fellow eye.

**Fig. 2:**
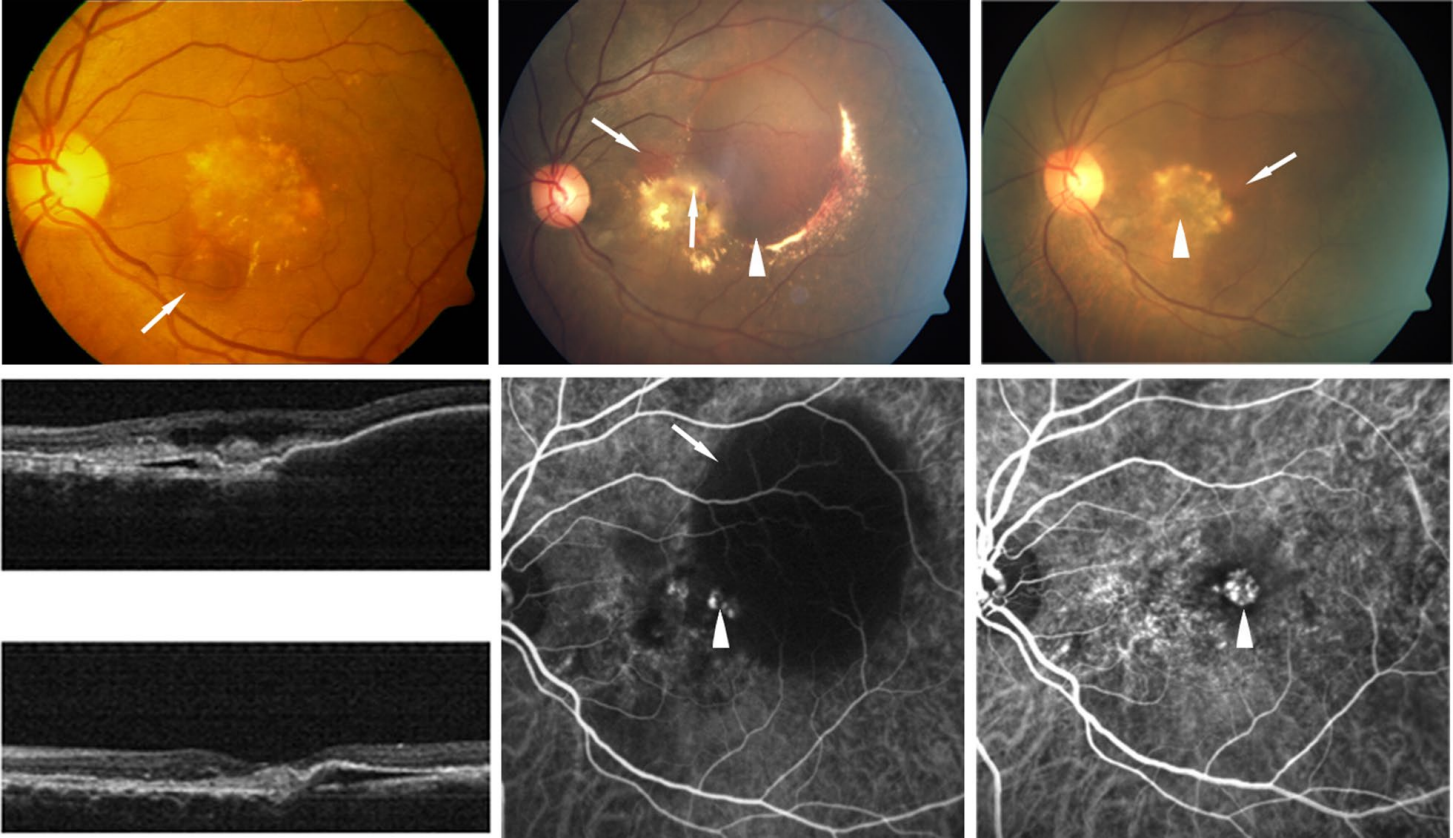
*Top* *row* shows polypoidal lesions (*white arrows*) demonstrated on indocyanine green angiography (ICG-A) in three study eyes done postoperatively after clearing of vitreous hemorrhage. *Bottom row* shows ICG-A of fellow eyes in three cases where the ocular media in the study eyes did not clear postoperatively to allow an angiography and the basis of diagnosis was demonstration of polypoidal lesions (*white arrows*) and/or choroidal branching vascular network (*white arrow heads*) in the fellow eye

**Fig. 3:**
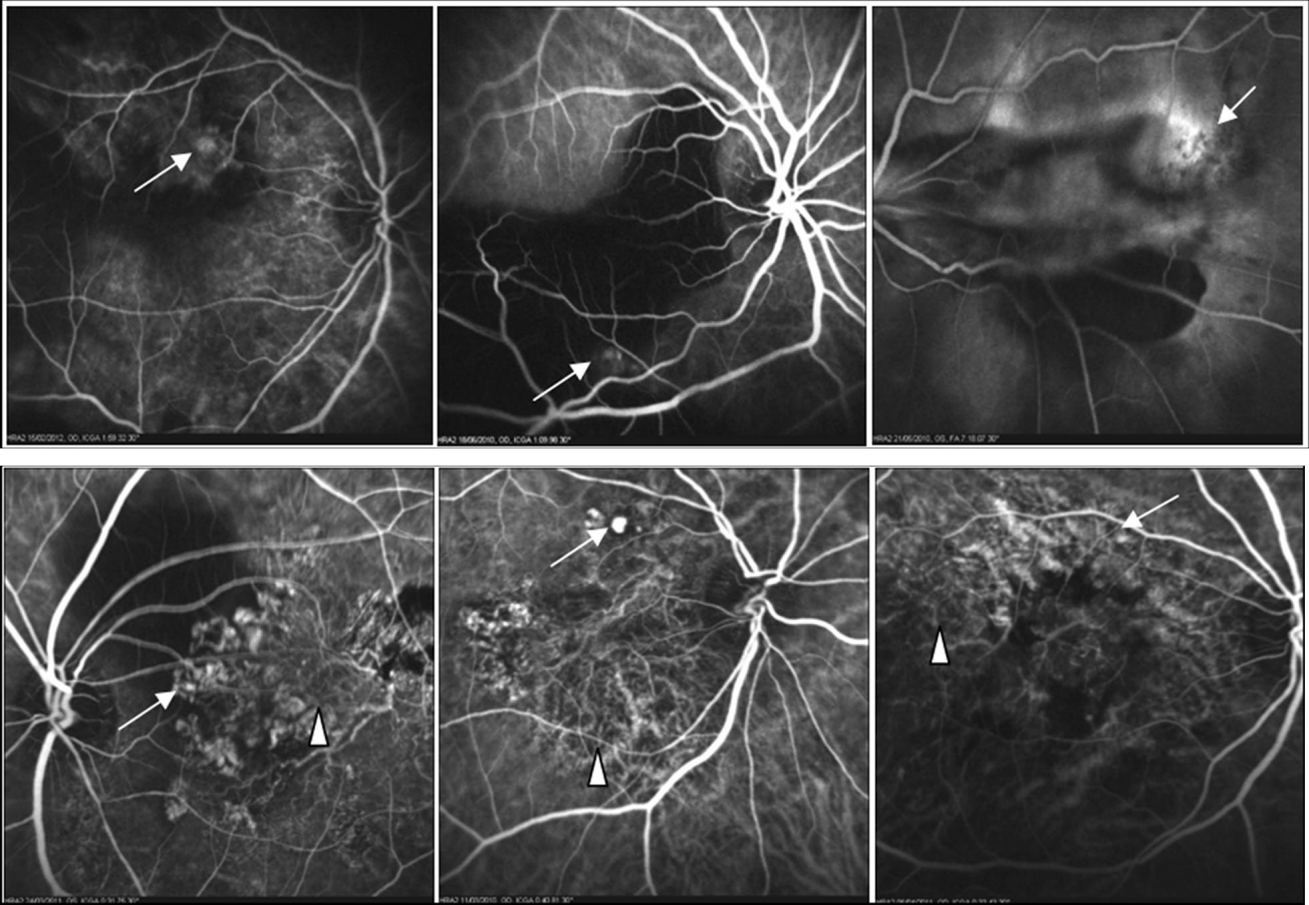
Both eyes of a 60 year old male with bilateral polypoidal choroidal vasculopathy (PCV) associated with vitreous hemorrhage (VH). *Left* *top picture* shows the fundus picture of the left eye at baseline before VH showing serous pigment epithelial detachment (PED, *white arrow*) and subretinal old blood. *Top centre picture* shows hemorrhagic pigment epithelial detachment (*white arrowhead*) and elevated orange yellow lesion before VH (*white arrow*). *Top right picture* shows fundus of the left eye post vitrectomy showing macular scar (*white arrowhead*), disappearance of previously seen polyp and development of a new orange elevated lesion (*white arrow*). *Middle right* indocyanine green angiography (ICG-A) of the left eye shows polypoidal lesions (*white arrowheads*) before VH. *Middle centre* shows ICG-A of the left eye after vitrectomy for VH shows disappearance of polyps and scar alone (*white arrowhead*). *Middle right* shows preoperative and postoperative OCT showing improvement of PED and resolution of intraretinal fluid. *Bottom right* fundus picture shows PED (*white arrowhead*). *Bottom centre* shows ICG angiography done before development of VH shows a polypoidal lesion (*white arrow*) and a PED (*white arrowhead*) and *bottom left picture* shows serosanguinous retinal detachment (*arrow*)

At presentation, fundus evaluation was not possible due to dense vitreous haemorrhage in 21 of the 28 eyes (75 %). B-scan showed vitreous haemorrhage in all 28 eyes, extensive exudative retinal detachment in six and suspected mass lesion in one eye. Diagnosis of underlying cause being PCV was based on demonstration of polyps or branching vascular on previous or post operative ICG-Angiography (ICG-A) in seven study eyes, four fellow eyes (Fig. 2) and on fluorescein angiography in two study eyes. The rest of the cases were diagnosed on the basis of presence of multiple sero-sanguinous RPE detachments suggestive of PCV. Two of the cases where polyps had been demonstrated on ICG-A before VH had regression of the previously noted polyps, but developed polyps in new locations subsequently (Fig. 3).

The site of subretinal haemorrhage was found to be predominantly macular in 12 (42.8 %) eyes, peripapillary in 4 (14.2 %) eyes, midperipheral in 10 (35.7 %) eyes and peripheral in 1 (3.5 %) eye. Intraoperatively, macular scar was noted in 9 (32.1 %) eyes and organized dehemoglobinized blood at the macula was noted in seven (25 %) eyes.

The most common surgical complication was iatrogenic retinal break which was seen in 5 (17.8 %) eyes, which occurred typically during induction of posterior vitreous detachment (PVD). Postoperatively, 10 (35.7 %) eyes developed visually significant cataract, 3 (10.7 %) eyes developed recurrent vitreous haemorrhage, 5 (17.8 %) had retinal detachment and 1 (3.5 %) patient had choroidal detachment. The mean IOP at baseline was 13.64 ± 2.52 and 13.57 ± 4.12 mm Hg at the last follow up.

## Discussion

Several reports and case series describing the diverse clinical spectrum, natural course, and role of various modalities of treatments for exudative PCV have been published in the last three decades [1, 2, 6–9, 11, 12], but there are few reports in the literature on the management of vitreous haemorrhage associated with PCV [6, 8, 9, 13]. Vitreous haemorrhage associated with PCV differs from other manifestations of PCV as these eyes are not amenable to ICG angiography. Unless the affected eye has been previously diagnosed as PCV or the fellow eye has features of PCV, it is difficult to diagnose them. B-scan features that can differentiate vitreous haemorrhage secondary to PCV from other causes has been reported and are very useful in this situation. B-scan can demonstrate defining features like blood lining an elevated membrane, hemorrhagic pigment epithelial detachment and absence of any acoustic shadowing. The natural course or role of any treatment modality is unknown for vitreous haemorrhage in PCV as no large series of cases has been studied.

In our study, PCV was bilateral in 9 (33.3 %) of which four eyes (14.8 %) also had PCV associated vitreous haemorrhage in the fellow eye. Previously, bilaterality has been reported ranging from 9 to 47 % for exudative PCV [1, 8, 9, 12, 14]. PCV had been originally reported in hypertensive middle aged African women [8, 14–16]. Associated systemic hypertension has been reported in 18–88 % in various case series and clinic pathological studies of PCV [8, 9, 14]. In our series, 59 % of the patients had systemic hypertension.

Vitreous haemorrhage associated with PCV has also been reported to occur following PDT [7, 17]. In our series only one patient had history of recent PDT and developed sudden onset loss of vision and VH 1 week after the treatment.

The earliest descriptions of vitreous haemorrhage in PCV was in 1985 [9, 10]. In previous reports by Kleiner et al. and Perkovich et al. [8, 9], visual recovery after vitrectomy was found to be equivocal. Two of the three eyes gained useful vision in one series [8], whereas the other studies reported worsening or no change in the visual acuity [4, 9]. These studies had small series ranging from one to three eyes that underwent vitrectomy. Recent reported outcomes of vitrectomy in PCV did not find a higher incidence of retinal breaks [13, 18].

In our study, significant visual improvement occurred in 16 of 29 eyes (57.1 %). Three patients recovered a visual acuity of better than 20/40, one eye had a visual acuity of 20/20 at 84 months, another had 20/25 at 17 months and the third eye had 20/40 at 6 months follow up. The improvement in visual acuity could have been limited by extensive blood under the macula. We did not attempt to remove the subretinal blood in our study. All cases in our study would have required subretinal tissue plasminogen activator with incubation of approximately half an hour. In our experience, the blood decolorizes rapidly in these cases and does not liquefy with conventional doses of tPA. In such cases, the dose of tPA is much more than the recommended upper limit of 50 μg. Further, washing of blood in such massive hemorrhage under the macula tends to cause photoreceptor damage. The peripheral visual field would also have improved after the clearance of vitreous haemorrhage but would be difficult to document in most cases due to poor central fixation.

The commonest intra operative complication was iatrogenic retinal breaks, which occurred during induction of PVD which was also been reported by us in our previous study [6]. Hence it is possibly better to wait for spontaneous PVD to occur before attempting surgery. The cases which had no visual recovery or experienced worsening of vision had macular scar, recurrent VH, post operative retinal detachment and cataract.

In a few cases in our study, polypoidal lesions which were seen on ICG-A before vitreous haemorrhage were not seen postoperatively but new lesions polypoidal lesions developed in a new location on subsequent follow up (Fig. 3). This disappearance of classic polypoidal lesions and replacement by RPE atrophy and scarring has been described in previously reported cases [1, 9]. The development of the haemorrhagic PEDs from the pre-existing polypoidal lesions, followed by breakthrough VH and then subsequent scarring may be the natural course of the condition.

## Conclusion

Our study shows that vitrectomy can produce significant visual improvement in patients with vitreous haemorrhage due to PCV. The incidence of retinal breaks during vitrectomy is high in PCV, and caution should be exercised while inducing PVD in such cases.

## Abbreviations

AMD: age-related macular degeneration
BCVA: best-corrected visual acuity
ETDRS: early treatment diabetic retinopathy study
ICGA: indocyanine angiography
IOP: intraocular pressure
PPV: pars plana vitrectomy
PL: perception of light
PDT: photo dynamic therapy
PED: pigment epithelial detachments
PCV: polypoidal choroidal vasculopathy
PVD: posterior vitreous detachment
VH: vitreous hemorrhage
